# Beyond General Prosocial Behavior: Nature Connectedness, Authenticity, and Instrumental Prosocial Behavior in Late Childhood

**DOI:** 10.3390/bs16101785

**Published:** 2026-10-01

**Authors:** Yaohua Zhang, Xiaohui Xu, Song Chang, Yunyun Huang, Min Xu, Sufei Xin

**Affiliations:** College of Education, Collaborative Innovation Center for the Mental Health of Youth from the Era of Conversion of New and Old Kinetic Energy Along the Yellow River Basin, Provincial Experimental Teaching Demonstration Center of Psychology, Ludong University, Yantai 264025, China; zhangyh@ldu.edu.cn (Y.Z.); xuxh3273@ldu.edu.cn (X.X.); songchang@ldu.edu.cn (S.C.);

**Keywords:** nature connectedness, authenticity, general prosocial behavior, instrumental prosocial behavior, late childhood

## Abstract

Research linking nature connectedness to prosociality has rarely distinguished motivationally specific forms of helping. The present study examined the associations of nature connectedness with general prosocial behavior and the more specific instrumental prosocial behavior during late childhood, with authenticity examined as an associational variable. Using a cross-sectional design, the final analytic sample comprised 281 Grade 4 and Grade 5 students aged 9–12 years who were recruited through convenience sampling from two primary schools in Yantai, China. Path analyses using factor scores adjusted for differential item functioning were interpreted within an Associational Variable Analysis framework. Nature connectedness was positively associated with authenticity and general prosocial behavior, with a significant positive indirect association involving authenticity. In contrast, authenticity was negatively associated with instrumental prosocial behavior. Nature connectedness showed a positive direct association with instrumental prosocial behavior after accounting for authenticity but a negative indirect association involving authenticity, resulting in a nonsignificant total association. The positive direct association differed by sex and was significant among boys but not girls. These findings underscore the importance of distinguishing instrumental from general prosocial behavior and suggest that authenticity may help differentiate their associations with nature connectedness in late childhood.

## 1. Introduction

Prosocial behavior is broadly defined as voluntary actions intended to benefit another individual or group (Eisenberg et al., 2015; Penner et al., 2005). It can manifest in various forms, including comforting, helping, and sharing. Due to its beneficial implications for both individual and societal development, prosocial behavior has attracted considerable attention from researchers across multiple disciplines (Hamburg & Hamburg, 2004; Memmott-Elison et al., 2020; Taylor, 2020). The importance of prosocial behavior is particularly evident during late childhood, a developmental period when children’s social relationships and adaptive functioning undergo substantial changes. Children’s levels of prosocial behavior have been shown to predict subsequent peer acceptance, social adjustment, and academic performance in adolescence (Cirimele et al., 2025; Oberle et al., 2023; Zhou et al., 2026), thereby serving as a protective factor against adolescent vulnerability (Crone & Achterberg, 2022; Hirani et al., 2022). Prosocial development is shaped by multiple interacting influences, including biological factors, socialization experiences, socioemotional competencies, and broader contextual processes (Malti & Dys, 2018). Identifying individual characteristics that may foster prosocial development is therefore an important objective in developmental research.

One potentially important characteristic is nature connectedness, referring broadly to individuals’ sense of identification and relational connection with the natural world. Research on positive human–nature relationships initially focused largely on their implications for environmental sustainability and pro-environmental behavior (Hughes et al., 2018; Ives et al., 2017; Tam, 2013). However, growing evidence indicates that nature connectedness is also associated with psychological well-being and adaptive functioning (Barragan-Jason et al., 2023; Pritchard et al., 2020). More recently, attention has begun to extend beyond individual well-being toward the interpersonal consequences of human–nature connectedness. A stronger connection with nature may foster a greater orientation toward the wider social world, potentially encouraging engagement with and responsiveness to others. Consistent with this possibility, accumulating evidence links nature connectedness with prosociality (Yao et al., 2025). However, this literature has generally treated prosocial behavior as a broad, undifferentiated construct, leaving unclear whether nature connectedness is similarly related to prosocial behavior when helping serves more explicitly instrumental or self-oriented goals.

This distinction is particularly relevant during late childhood, when the motivational bases of prosocial behavior become increasingly differentiated. As children develop, helping may reflect not only concern for others or adherence to social expectations but also strategic considerations such as gaining approval, eliciting reciprocity, enhancing social standing, or maintaining desirable relationships (Grueneisen & Warneken, 2022). Instrumental prosocial behavior represents a more specific instance of this motivational complexity, in which benefiting others also serves self-oriented goals (Boxer et al., 2004). Late childhood is marked by increasing sensitivity to peer evaluation and social acceptance (LaFontana & Cillessen, 2010), making the relationship between externally oriented social motives and children’s emerging sense of self especially salient. Authenticity may therefore be particularly relevant to understanding how nature connectedness relates to prosociality during this developmental period. Nature connectedness may broaden children’s social orientation and facilitate greater alignment with their internally endorsed values and experiences. These processes may have different implications for general prosocial behavior and the more specific instrumental prosocial behavior.

Accordingly, the present study examined two related questions during late childhood: (1) whether nature connectedness was associated with general prosocial behavior and the more specific instrumental prosocial behavior; and (2) whether authenticity was differentially associated with these outcomes.

### 1.1. The Link Between Nature Connectedness and Prosociality

Nature connectedness has increasingly been recognized as an important aspect of children’s and adolescents’ development, as it reflects individuals’ fundamental relationship with the natural world and is associated with a range of adaptive psychological and social outcomes (Harris et al., 2025; Yildirim et al., 2026). Although nature connectedness has been conceptualized in various ways across different theoretical perspectives (Clayton, 2003; C. Martin & Czellar, 2016; Mayer & Frantz, 2004; Nisbet et al., 2009; Schultz, 2002), the present study adopts the definition proposed by Salazar et al. (2020), who conceptualized nature connectedness as “the way people identify with predominantly natural landscapes and the relationships they form with the elements in those environments.” This definition emphasizes that nature connectedness involves not only an individual’s subjective attachment to nature but also a sense of identification and relational connection with entities beyond the self. Such a broadened sense of connectedness may contribute to the development of a more expanded and self-transcendent sense of identity (Olivos & Clayton, 2017), which may subsequently shape how individuals relate to others. At the interpersonal level, this expanded sense of identity may manifest as a broader social-relational orientation, characterized by greater attention to the social world and a stronger sense of connection with others. This social-relational orientation may, in turn, promote greater attention to others and more active engagement in interpersonal and collective contexts (Arbuthnott, 2023; Goldy & Piff, 2020). In this sense, the social consequences of nature connectedness may encompass a broader tendency to attend to, connect with, and respond to other people, thereby increasing individuals’ general propensity to engage in prosocial behavior. Consistent with this account, a recent meta-analysis demonstrated a robust positive association between nature connectedness and prosociality (Yao et al., 2025), suggesting that individuals who feel more connected with nature are also more likely to engage in behaviors that benefit others.

Nevertheless, research linking nature connectedness to prosociality has largely relied on broad measures of prosocial behavior, leaving its associations with more motivationally specific forms of helping less understood. Prosocial behavior encompasses diverse forms of helping that may differ in their underlying motivations (Böckler et al., 2016; Eisenberg et al., 2016; A. Martin & Olson, 2015). Conventional measures of general prosocial behavior typically assess whether individuals engage in helping, sharing, or caring behaviors without specifying the motives underlying these actions. Thus, such behaviors may reflect genuine concern for others, adherence to social norms, expectations of reciprocity, self-serving considerations, or some combination of these motives. The motivational bases of prosocial behavior become increasingly differentiated across childhood (Grueneisen & Warneken, 2022; Hepach & Daniel, 2026). One important developmental manifestation of this differentiation is the emergence of instrumental prosocial behavior, whereby children use helping strategically as a means to achieve ulterior self-serving or self-benefiting goals (Boxer et al., 2004; Grueneisen & Warneken, 2022). Unlike broad measures of prosocial behavior that leave the underlying motive unspecified, instrumental prosocial behavior explicitly incorporates a self-oriented motivational component, as helping may serve goals such as gaining approval, enhancing social standing, eliciting reciprocity, or establishing desirable social relationships (Carlo & Padilla-Walker, 2020; Eisenberg et al., 2015). Boxer et al. (2004) originally labeled this construct proactive prosocial behavior. Although the term proactive can also be understood as referring to self-initiated or anticipatory action, Boxer et al. used it specifically to denote the instrumental, goal-directed function of the behavior. Consistent with this functional emphasis, subsequent research has referred to this construct as instrumental prosocial behavior (Mouratidis et al., 2019), and developmental accounts likewise describe children’s strategic prosocial acts as instrumental means of pursuing further goals (Grueneisen & Warneken, 2022). Accordingly, the present study uses instrumental prosocial behavior as a theoretically descriptive label because it more directly conveys the defining motivational feature captured by the items—benefiting others as a means of advancing self-oriented goals. Although this terminology emphasizes the self-oriented motivational component of such behavior, it does not imply that instrumental prosocial behavior is necessarily cynical or purely selfish, because it can facilitate mutually beneficial cooperation and help individuals initiate and navigate social relationships (Grueneisen & Warneken, 2022). This dual nature makes instrumental prosocial behavior particularly relevant to a social-relational account of nature connectedness. Although instrumental helping may serve self-interest, it still entails engagement with others and can function to initiate, maintain, or strengthen social relationships. Accordingly, the broader social orientation associated with nature connectedness may be positively associated with general prosocial behavior and may also favor engagement in the more specific instrumental prosocial behavior, given that the latter can serve important interpersonal and relational functions. Yet, such a shared social-relational tendency need not translate into parallel overall associations, because nature connectedness may simultaneously be associated with psychological variables that have different implications for general and instrumental prosocial behavior.

### 1.2. The Associational Role of Authenticity

One such associational variable is authenticity, which may be particularly relevant given the different motivational characteristics of general and instrumental prosocial behavior. Kernis and Goldman (2006) defined authenticity as “the unobstructed operation of one’s true- or core-self in one’s daily enterprise.” This conceptualization emphasizes that authenticity involves the subjective experience of being one’s true, unvarnished self, in which individuals perceive their thoughts, feelings, and behaviors as consistent with their core selves (Alchin et al., 2024; Sedikides & Schlegel, 2024; Wood et al., 2008). The expanded and self-transcendent sense of identity associated with nature connectedness may also have implications for authenticity. At the intrapersonal level, reaching beyond one’s self-boundary and experiencing greater connectedness with a broader context may create psychological distance from narrow self-concerns and external expectations (Davis & Gatersleben, 2013; Mei et al., 2024; Ruckert et al., 2025). Such self-transcendence may facilitate greater awareness of personally meaningful values and goals, allowing individuals to orient their behavior toward what they genuinely endorse. Consistent with this account, Jiang and Sedikides (2022) proposed and demonstrated that self-transcendence promotes authentic-self pursuit. Nature connectedness may therefore enhance individuals’ sense of authenticity by fostering a self-transcendent orientation that facilitates greater alignment with their inner values and experiences.

Although authenticity has traditionally been examined primarily in relation to individual well-being and psychological functioning (Alchin et al., 2024; Sedikides & Schlegel, 2024), emerging perspectives suggest that its significance extends beyond the self to interpersonal relationships and prosocial engagement. Its emphasis on self-congruence provides an important basis for understanding how authenticity may relate to prosocial behavior. Jiang and Sedikides (2022) proposed that authentic-self pursuit orients individuals toward behavior that is consistent with their pivotal values and demonstrated across multiple studies that authentic-self pursuit was positively associated with general prosociality but negatively associated with prosociality that conflicted with one’s authentic self. Related interpersonal evidence indicates that authenticity is positively associated with compassionate goals and constructive interpersonal strategies, but negatively associated with self-image goals concerned with obtaining others’ approval and maintaining a desired social image (Bayır-Toper et al., 2022; Tou et al., 2015). Developmental research further shows that authenticity in peer relationships predicts greater prosocial experiences among adolescents (Theran, 2021). Together, these findings suggest that authenticity has meaningful implications not only for individuals’ inner functioning but also for how they orient themselves and behave toward others.

These implications, however, may differ for general and instrumental prosocial behavior. Given its motivationally unspecified nature, general prosocial behavior may broadly align with authentic self-expression without necessarily reflecting altruistic motivation (Jiang & Sedikides, 2022; Theran, 2021). Instrumental prosocial behavior, by contrast, explicitly involves self-oriented goals that conceptually overlap with the self-image and externally oriented concerns less characteristic of authentic functioning (Jiang & Sedikides, 2022; Tou et al., 2015). Accordingly, authenticity may be positively associated with general prosocial behavior while being negatively associated with the more specific instrumental prosocial behavior.

### 1.3. The Present Study

Building on the perspective that nature connectedness may foster a more expanded and self-transcendent sense of identity (Olivos & Clayton, 2017), the present study examined how nature connectedness relates to general prosocial behavior and the more specific instrumental prosocial behavior during late childhood. Late childhood is a particularly relevant developmental period because children’s prosocial behavior becomes increasingly differentiated in its motivational bases, including the growing capacity to use helping strategically in pursuit of self-oriented goals (Grueneisen & Warneken, 2022; Hepach & Daniel, 2026). Although previous research has demonstrated a positive association between nature connectedness and prosociality (Yao et al., 2025), research examining this association has typically relied on broad measures of prosocial behavior. Consequently, little is known about whether nature connectedness is similarly associated with instrumental prosocial behavior, in which helping also serves self-oriented goals.

The present study further examined authenticity as an associational variable that may help clarify these associations. An expanded and self-transcendent sense of identity associated with nature connectedness may facilitate authentic-self pursuit by helping individuals move beyond narrow self-concerns and orient themselves toward personally meaningful values and goals (Jiang & Sedikides, 2022). Authenticity, in turn, may have different implications for general and instrumental prosocial behavior. Greater alignment with the authentic self has been associated with general prosociality, whereas authentic functioning is less characteristic of behavior guided by self-image and externally oriented concerns (Jiang & Sedikides, 2022; Tou et al., 2015). Thus, authenticity may be positively associated with general prosocial behavior while being negatively associated with prosocial behavior instrumentally for self-oriented purposes.

Accordingly, we hypothesized that nature connectedness would be positively associated with authenticity, which in turn would be positively associated with general prosocial behavior but negatively associated with instrumental prosocial behavior. We further expected nature connectedness to be positively associated with general prosocial behavior. For instrumental prosocial behavior, however, we anticipated potentially countervailing associations. The broader social orientation associated with nature connectedness may favor instrumental prosocial engagement, whereas the indirect association involving authenticity was expected to be negative. Thus, no directional hypothesis was specified for the overall association between nature connectedness and instrumental prosocial behavior. Given limited prior evidence regarding sex differences in these associations, exploratory multigroup analyses were also conducted to examine whether the structural associations differed between boys and girls, without specifying directional hypotheses.

## 2. Materials and Methods

### 2.1. Participants and Procedure

A total of 295 students participated in the study. They were recruited through convenience sampling from two public primary schools in Yantai, a coastal city in Shandong Province, China. The schools were selected based on accessibility and willingness to participate and were located in urban areas, primarily serving children from the surrounding communities. Following school approval, eight Grade 4 and Grade 5 classrooms were selected in coordination with school administrators, and all students enrolled in these classrooms were invited to participate. Participation required written parental consent and the child’s assent. However, complete records of the total number of eligible or invited students were not retained for the study; therefore, a precise participation rate could not be calculated. Of the 295 participants, 83 attended School 1 and 212 attended School 2. Detailed indicators of family socioeconomic status were not collected because the participating schools expressed concerns that collecting such information might encourage sensitive social comparisons among students and raise privacy concerns. After data screening, 14 participants were excluded due to problematic response patterns identified by long strings of identical consecutive responses. The final analytic sample consisted of 281 students, including 148 fourth-grade students (52.7%) and 133 fifth-grade students (47.3%). There were 148 boys (52.7%) and 133 girls (47.3%). Participants ranged in age from 9 to 12 years (*M* = 10.31, *SD* = 0.68).

Before data collection, informed consent was obtained from parents or legal guardians, and students provided their assent to participate. Participants were informed that participation was voluntary and that they could withdraw at any time without any negative consequences. The questionnaires were administered collectively in classrooms under the supervision of trained researchers. Teachers were not involved in viewing or evaluating students’ responses. All responses were collected anonymously, and confidentiality was ensured throughout the study. This study was conducted in accordance with the Declaration of Helsinki and approved by the Ethics Review Committee of the College of Education, Ludong University.

### 2.2. Measures

**Nature Connectedness.** Nature connectedness was assessed using the Connection to Nature Index developed by Cheng and Monroe (2012), which was originally designed to measure nature connectedness among 9- to 10-year-old children. The scale was later validated in Taiwan and revised into a 14-item version with an expanded age range of 8–12 years (Salazar et al., 2021). For the present study, the wording of this version was adapted to Simplified Chinese while retaining the original item meanings. Participants rated each item on a 5-point scale (1 = do not like at all, 5 = like very much). An example item is “Feel comfortable and peaceful in nature.” Confirmatory factor analysis supported the hypothesized single-factor structure, χ^2^(77) = 152.25, CFI = 0.94, TLI = 0.93, RMSEA = 0.06, and SRMR = 0.05. In the present study, Cronbach’s α = 0.93 and McDonald’s ω = 0.95.

**Authenticity.** Authenticity was assessed using the 12-item Authenticity Scale developed by Wood et al. (2008). Although the scale was initially developed for adults, its applicability has subsequently been validated among adolescents (Thomaes et al., 2017), and the Chinese version validated by Song et al. (2020) was used in the present study. The scale consists of three dimensions: self-alienation, authentic living, and accepting external influence, with the self-alienation and accepting external influence items reverse-scored so that higher scores consistently reflected greater authenticity. These dimensions capture the extent to which individuals perceive themselves as being in touch with and acting according to their true selves, while being relatively independent from others’ views and influences. Participants rated each item on a 5-point scale ranging from 1 (does not describe me at all) to 5 (describes me very well). An example item is “I am true to myself in most situations.” Confirmatory factor analysis supported the hypothesized correlated three-factor structure, χ^2^(48) = 62.58, *p* = 0.077, CFI = 0.98, TLI = 0.98, RMSEA = 0.04, and SRMR = 0.05. In the present study, Cronbach’s α = 0.82 and McDonald’s ω = 0.87.

**General Prosocial Behavior.** General prosocial behavior was assessed using the prosocial behavior subscale of the self-report version of the Strengths and Difficulties Questionnaire (Goodman et al., 1998). We used the previously validated Chinese version, which has demonstrated satisfactory psychometric properties among Chinese preadolescents and adolescents (Du et al., 2008). The prosocial behavior subscale consists of five items assessing prosocial tendencies, including being considerate, sharing, caring, kindness, and helping. Participants rated their behaviors over the past six months on a 3-point scale (1 = not true, 2 = somewhat true, 3 = certainly true). An example item is “I care about their feelings.” Confirmatory factor analysis supported the hypothesized single-factor structure, χ^2^(5) = 2.49, CFI = 1.00, TLI = 1.00, RMSEA = 0.00, and SRMR = 0.02. In the present study, Cronbach’s α = 0.71 and McDonald’s ω = 0.72.

**Instrumental Prosocial Behavior.** Instrumental prosocial behavior was assessed using the five-item Proactive Prosocial subscale of the Aggressive and Prosocial Behavior Questionnaire (Boxer et al., 2004). Although Boxer et al. labeled the subscale proactive prosocial behavior, its items were designed to assess instrumental, goal-directed prosocial actions; accordingly, instrumental prosocial behavior is used as the theoretical label in the present study. For use with the present Chinese sample, the items were translated into Chinese and back-translated into English following Brislin’s (1970) procedure. Discrepancies between the original and back-translated versions were discussed and resolved to enhance semantic and conceptual correspondence. The five items assess helping, sharing, lending, doing favors, and complimenting others when these behaviors are used to obtain a desired outcome (e.g., “I often help people to get what I want”). Confirmatory factor analysis supported the hypothesized single-factor structure, χ^2^(5) = 7.92, CFI = 0.99, TLI = 0.98, RMSEA = 0.05, and SRMR = 0.03. In the present study, Cronbach’s α = 0.89 and McDonald’s ω = 0.90.

### 2.3. Data Analysis Plan

All analyses were conducted in R 4.5.3 (R Core Team, 2026). Data preprocessing was performed using the tidyverse package (Wickham et al., 2019), with long-string response patterns screened using the long-string index implemented in the careless package (Yentes & Wilhelm, 2023). Participants with eight or more consecutive identical responses were excluded (n = 14; Maniaci & Rogge, 2014). Item-level missingness was minimal, with the proportion of missing responses not exceeding 2.85% for any item. Missing responses were imputed using the participant’s mean score across the remaining items of the corresponding scale. Descriptive statistics and Pearson correlations were then calculated, and scale reliability was assessed using Cronbach’s α and McDonald’s ω with the psych package (Revelle, 2026). The structural validity of each measure was examined using confirmatory factor analysis (CFA) with the robust maximum likelihood (MLR) estimator in the lavaan package (Rosseel, 2012). Model fit was evaluated using the comparative fit index (CFI), Tucker–Lewis index (TLI), root mean square error of approximation (RMSEA), and standardized root mean square residual (SRMR). Following commonly used guidelines for evaluating model fit (Kline, 2016), CFI and TLI values ≥ 0.90 and RMSEA and SRMR values ≤ 0.08 were considered indicative of acceptable model fit.

Preliminary multigroup CFA suggested potential measurement noninvariance across sex, particularly for general prosocial behavior, for which metric invariance was not established. Regularized moderated nonlinear factor analysis (MNLFA) was therefore conducted using the regDIF package (Belzak, 2023) to more flexibly evaluate and account for potential measurement bias. Compared with traditional multigroup approaches, MNLFA allows multiple categorical and continuous background variables to be examined simultaneously and can model differential item functioning in both item intercepts and factor loadings (Bauer et al., 2020; Belzak & Bauer, 2020). This approach has also been increasingly adopted in recent empirical research (Behrens et al., 2025; Magro et al., 2024, 2026; Stevens et al., 2022). In MNLFA, effects of background variables on the latent factor mean and variance are termed impact and represent covariate-related differences in the latent distribution, whereas effects on item intercepts and factor loadings represent differential item functioning (DIF). Moderation of item intercepts indicates uniform DIF, whereas moderation of factor loadings indicates nonuniform DIF. Simulation studies have shown that LASSO-regularized MNLFA provides better control of Type I error rates while maintaining comparable power to detect true DIF effects relative to traditional DIF-testing procedures (Bauer et al., 2020). In the present study, regularized MNLFA models were estimated separately for each scale or subscale using the regDIF package (Belzak, 2023). Specifically, sex (boys = −1, girls = 1) and grade (Grade 4 = −1, Grade 5 = 1) were effect-coded and entered simultaneously as predictors in both the latent-factor impact equations and the item-level DIF equations. Item responses were treated as continuous indicators using the CFA specification (item.type = “cfa”), and the predictors were internally standardized using the default setting of regDIF. No anchor items were prespecified. The default LASSO penalty was applied to the candidate intercept and loading DIF parameters across 100 automatically generated values of the tuning parameter, and the solution with the lowest Bayesian information criterion (BIC) was selected. Consistent with the preliminary multigroup CFA results, the BIC-selected regularized MNLFA solution retained both intercept and loading DIF effects for general prosocial behavior as well as authentic living only. For general prosocial behavior, the optimal model retained 14 nonzero DIF parameters, comprising seven intercept effects representing uniform DIF and seven loading effects representing nonuniform DIF. Eight of these effects were associated with grade and six with sex. For authentic living, one sex-related intercept DIF effect was retained for Item 8. No nonzero DIF parameters were retained for instrumental prosocial behavior, nature connectedness, self-alienation, or accepting external influence. Complete model-selection information and penalized DIF estimates are reported in Table A1 in the Appendix A. Based on the final models, person-specific expected a posteriori (EAP) factor scores incorporating the identified DIF effects were obtained. Because the regDIF approach estimates unidimensional factor models, the three dimensions of authenticity—self-alienation, authentic living, and accepting external influence—were analyzed separately. DIF-adjusted EAP factor scores were obtained for each dimension and oriented so that higher scores consistently reflected greater authenticity. The three dimension scores were then averaged to create an overall authenticity score. For the remaining measures, DIF-adjusted EAP factor scores were obtained directly from their respective MNLFA models. These scores accounted for systematic differences in item functioning associated with sex and grade and were used in all subsequent substantive analyses.

Given the nonexperimental cross-sectional design, the structural analyses were framed using Weems’ (2025) Associational Variable Analysis (AVA), a conceptual framework for studies with mediation-type aims. Because cross-sectional data cannot establish temporal ordering or causal direction, a putative mediator cannot be interpreted as a temporally ordered mechanism; instead, it is treated as an associational variable that may statistically account for part of an observed association. AVA retains the analytic techniques developed for mediation but interprets the resulting estimates in associational rather than causal terms. Accordingly, authenticity was treated as an associational variable rather than a mediator, and the resulting estimates are described as total, direct, and indirect associations rather than effects. Following the AVA framework, path analyses were conducted using the DIF-adjusted EAP factor scores to examine the associations among nature connectedness, authenticity, general prosocial behavior, and the more specific instrumental prosocial behavior. Authenticity was specified as the associational variable in estimating the indirect associations of nature connectedness with general prosocial behavior and with the more specific instrumental prosocial behavior. The models were estimated using maximum likelihood (ML) in lavaan (Rosseel, 2012), and the indirect associations were evaluated using 95% bootstrap confidence intervals based on 5000 resamples. Finally, to explore potential group differences and evaluate the robustness of the structural associations, separate multigroup path analyses were conducted in lavaan to examine whether the structural path coefficients were equivalent across sex (boys vs. girls) and grade (Grade 4 vs. Grade 5).

## 3. Results

### 3.1. Common Method Variance Test

To assess the potential influence of common method bias, Harman’s single-factor test was conducted. All items from the study variables were entered into an exploratory factor analysis without rotation, and the proportion of variance explained by the first unrotated factor was examined. The results showed that the first unrotated factor accounted for 25.10% of the total variance. Although this result did not indicate a dominant single-factor structure, the limitations of Harman’s single-factor test mean that common method variance cannot be ruled out.

### 3.2. Descriptive Statistics and Correlation Analysis

The means, standard deviations, and correlations among the study variables are presented in Table 1. Correlations based on the raw mean scores are presented in the lower triangle and are reported below, whereas those based on the DIF-adjusted latent factor scores are presented in the upper triangle for comparison. Nature connectedness was positively correlated with authenticity (*r* = 0.28, *p* < 0.001) and general prosocial behavior (*r* = 0.40, *p* < 0.001), suggesting that children with greater connectedness to nature tended to report higher levels of authenticity and general helping behaviors. Authenticity was also positively associated with general prosocial behavior (*r* = 0.27, *p* < 0.001) and negatively associated with instrumental prosocial behavior (*r* = −0.21, *p* < 0.001). In contrast, nature connectedness was not significantly associated with instrumental prosocial behavior (*r* = 0.07, *p* = 0.22). General and instrumental prosocial behaviors were not significantly correlated with each other (*r* = 0.00, *p* = 0.96), which is consistent with previous findings by Boxer et al. (2004). This pattern indicates that instrumental prosocial behavior may represent a specific aspect of prosocial engagement that is not simply reflected by general prosocial tendencies. Regarding sex differences, girls reported higher levels of nature connectedness (*r* = 0.17, *p* = 0.01) and general prosocial behavior (*r* = 0.12, *p* = 0.05). However, sex was not significantly associated with authenticity or instrumental prosocial behavior. Importantly, the correlation pattern based on the DIF-adjusted latent factor scores (upper triangle) was largely consistent with that based on the raw mean scores (lower triangle), indicating that the substantive associations among the study variables were robust across different scoring approaches.

### 3.3. Associational Variable Analysis and Multigroup Path Analyses

To examine the hypothesized associational pattern involving authenticity, we estimated a path model in which nature connectedness was specified as the focal variable, authenticity as the associational variable, and general and instrumental prosocial behaviors as simultaneous outcomes. This ordering was used to parameterize the theoretically proposed model and was not interpreted as temporal or causal. The results are presented in Table 2. Nature connectedness was positively associated with authenticity (a path; β = 0.27, *p* < 0.001), and authenticity was positively associated with general prosocial behavior after accounting for nature connectedness (b_1_ path; β = 0.14, *p* = 0.03). Based on 5000 bootstrap resamples, the positive indirect association between nature connectedness and general prosocial behavior involving authenticity was significant (β_indirect_ = 0.04, *SE* = 0.02, 95% bootstrap CI [0.01, 0.08]). The direct association between nature connectedness and general prosocial behavior remained significant after accounting for authenticity (β = 0.37, *p* < 0.001), as did the total association (β = 0.41, *p* < 0.001).

For instrumental prosocial behavior, authenticity showed a significant negative association after accounting for nature connectedness (b_2_ path; β = −0.27, *p* < 0.001). The indirect association between nature connectedness and instrumental prosocial behavior involving authenticity was therefore significant and negative (β_indirect_ = −0.08, *SE* = 0.02, 95% bootstrap CI [−0.13, −0.03]). After accounting for authenticity, nature connectedness showed a significant positive direct association with instrumental prosocial behavior (β = 0.15, *p* = 0.02), whereas the total association was nonsignificant (β = 0.07, *p* = 0.24). Thus, the positive direct association and negative indirect association had opposing signs and largely offset one another. In conventional mediation terminology, such a pattern has been described as inconsistent mediation (MacKinnon et al., 2000) or competitive mediation (Zhao et al., 2010). Under the AVA framework, however, it is interpreted here as a pattern of countervailing direct and indirect associations rather than evidence of a mediational process. Consistent with the near-zero zero-order correlations between general and instrumental prosocial behavior based on both the raw mean scores (*r* = 0.00) and the DIF-adjusted EAP factor scores (*r* = −0.01), their residual covariance in the path model was also nonsignificant (standardized estimate = −0.003, *p* = 0.96).

To examine whether the structural paths differed by sex, a multigroup path analysis was conducted. The unconstrained model was saturated (χ^2^(0) = 0.00, CFI = 1.00, TLI = 1.00, RMSEA = 0.00, and SRMR = 0.00). A fully constrained model was then estimated by imposing equality constraints on all five structural paths across boys and girls. This model showed poorer fit: χ^2^(5) = 10.99, *p* = 0.05, CFI = 0.94, TLI = 0.85, RMSEA = 0.09, and SRMR = 0.06, with ΔCFI = −0.06 relative to the unconstrained model, suggesting that at least one structural path differed by sex. A Lagrange multiplier score test indicated that the path from nature connectedness to instrumental prosocial behavior differed significantly by sex (χ^2^(1) = 7.62, *p* = 0.01), whereas the remaining structural paths did not show significant sex differences (*p*s > 0.05). Accordingly, a partial structural invariance model was specified by freeing this path while imposing equality constraints on the remaining four structural paths. The partial-invariance model showed good fit: χ^2^(4) = 3.17, *p* = 0.53, CFI = 1.00, TLI = 1.03, RMSEA = 0.00, and SRMR = 0.04, with ΔCFI = 0.00 relative to the unconstrained model. These results supported invariance of the remaining structural paths across boys and girls.

Inspection of the group-specific estimates showed that the pattern of associations was largely similar across boys and girls, except for the direct association between nature connectedness and instrumental prosocial behavior. Because the component paths of the indirect associations were constrained to equality across groups, the corresponding unstandardized indirect associations were identical for boys and girls, although the standardized indirect associations could differ slightly because standardization was based on group-specific variances. For general prosocial behavior, the direct association was significant in both groups (boys: *B*_direct_ = 0.36, β_direct_ = 0.38, *p* < 0.001; girls: *B*_direct_ = 0.36, β_direct_ = 0.32, *p* < 0.001). The indirect association involving authenticity was also significant (*B*_indirect_ = 0.04, 95% bootstrap CI [0.01, 0.09]), corresponding to β_indirect_ = 0.04 for boys and β_indirect_ = 0.04 for girls. The total association was significant in both groups (*B*_total_ = 0.41, *p* < 0.001), corresponding to β_total_ = 0.42 for boys and β_total_ = 0.36 for girls.

For instrumental prosocial behavior, the direct association was positive and significant among boys (*B*_direct_ = 0.26, β_direct_ = 0.25, *p* = 0.003) but nonsignificant among girls (*B*_direct_ = −0.10, β_direct_ = −0.09, *p* = 0.36). The negative indirect association involving authenticity was significant in both groups (*B*_indirect_ = −0.08, 95% bootstrap CI [−0.13, −0.03]), corresponding to β_indirect_ = −0.07 for boys and β_indirect_ = −0.07 for girls. Consequently, the total association was positive and significant among boys (*B*_total_ = 0.18, β_total_ = 0.18, *p* = 0.047) but negative and nonsignificant among girls (*B*_total_ = −0.18, β_total_ = −0.15, *p* = 0.10). Thus, the observed sex difference was localized to the direct association between nature connectedness and instrumental prosocial behavior.

The same multigroup procedure was used to examine structural path invariance across grade. The unconstrained model was saturated and therefore fit the data perfectly by definition. Constraining all five structural paths to equality across Grade 4 and Grade 5 did not significantly worsen model fit (Δχ^2^(5) = 5.05, *p* = 0.41). The omnibus score test was also nonsignificant (χ^2^(5) = 5.00, *p* = 0.42), and none of the five path-specific score tests was significant (all *p*s ≥ 0.135). These findings supported full structural path invariance across grades. Accordingly, no paths were freed, and a partial-invariance model was not required.

## 4. Discussion

The present study examined whether nature connectedness was associated with general prosocial behavior and the more specific instrumental prosocial behavior during late childhood, and whether these associations included indirect associations through authenticity. By distinguishing instrumental prosocial behavior from general prosocial tendencies, the present study extends previous research that has largely relied on broad measures of prosocial behavior. Three major findings emerged. First, nature connectedness showed positive total and direct associations with general prosocial behavior, together with a significant positive indirect association involving authenticity. Second, its association with the more specific instrumental prosocial behavior comprised a positive direct association and a significant negative indirect association involving authenticity. These countervailing associations largely offset one another, resulting in a nonsignificant total association. Third, the direct association between nature connectedness and instrumental prosocial behavior differed by sex: it was positive and significant among boys but nonsignificant among girls. The remaining structural paths were invariant across sex, and all five structural paths were invariant across Grade 4 and Grade 5.

### 4.1. Differential Associations of Nature Connectedness with General and Instrumental Prosocial Behavior

The present study extends previous research by showing that the association between nature connectedness and prosociality depends on how prosocial behavior is conceptualized. Consistent with previous research linking nature connectedness to prosociality (Yao et al., 2025), nature connectedness was positively associated with general prosocial behavior, and this association remained significant after accounting for authenticity. By contrast, the overall association between nature connectedness and the more specific instrumental prosocial behavior was nonsignificant. Notably, however, a positive direct association emerged after authenticity was taken into account. This pattern suggests that treating prosocial behavior as a single broad construct may conceal meaningful differences in how nature connectedness relates to prosocial behavior with more explicitly specified motivational characteristics.

The positive association with general prosocial behavior may be understood from an expanded-identity perspective (Olivos & Clayton, 2017). Its social-relational implications suggest that a broadened sense of self may extend into the interpersonal domain by increasing attention to and engagement with others (Arbuthnott, 2023; Goldy & Piff, 2020). Such an outward-oriented stance may foster a general tendency to respond positively to others and engage in helping, sharing, and caring behaviors. Importantly, this interpretation does not assume that general prosocial behavior is driven by any single motive. Rather, because general prosocial measures capture prosocial actions without specifying their underlying motivations, the observed positive association may reflect the broader social orientation and responsiveness to others associated with nature connectedness. The positive direct association with instrumental prosocial behavior can also be understood from the social-relational account. Although instrumental helping serves self-interest, it nevertheless involves engagement with others and may function to obtain reciprocity, strengthen social relationships, or secure desirable social outcomes. Strategic prosociality can therefore serve important interpersonal functions by facilitating mutually beneficial cooperation and helping children initiate and regulate social relationships (Grueneisen & Warneken, 2022). Nature connectedness may similarly foster greater engagement with the social world, making children more likely to participate in prosocial interactions even when such behavior also serves instrumental goals. The emergence of a positive direct association after accounting for authenticity is therefore consistent with the possibility that nature connectedness may have social-relational implications that favor instrumental prosocial engagement. At the same time, the absence of a significant overall association suggests that this positive tendency may coexist with psychological processes operating in the opposite direction, a possibility considered in greater detail in the following section.

The positive direct association between nature connectedness and instrumental prosocial behavior was primarily evident among boys. One tentative interpretation is that the social-relational implications of nature connectedness may be expressed differently between boys and girls. Previous work suggests that prosocial behaviors associated with agentic goals, social standing, and popularity may be particularly relevant to more instrumental forms of helping (Findley-Van Nostrand & Ojanen, 2018), while broader gender-role perspectives propose that boys and men may be more likely to express prosociality through agentic or goal-directed forms of social behavior (Eagly, 2009). Nature connectedness may therefore be more readily translated into instrumental prosocial engagement among boys when helping also serves interpersonal or status-related goals. However, because the overall level of instrumental prosocial behavior did not differ by sex and the present study did not directly assess the proposed motivational processes, this interpretation should be regarded as tentative and requires replication.

An alternative, though complementary, explanation may be offered by the moral expansiveness framework. From this perspective, an expanded and self-transcendent sense of identity may broaden the boundaries of individuals’ moral community, leading them to extend care and moral consideration beyond the self (Crimston et al., 2016; Singer, 2011). This account may be particularly relevant to the positive association between nature connectedness and general prosocial behavior, insofar as a broader moral orientation, characterized by an expanded circle of moral concern, may increase individuals’ general willingness to respond to and benefit others. Importantly, however, the present measure of general prosocial behavior does not specify the motives underlying helping; thus, the observed association cannot be interpreted as evidence that such behavior was necessarily driven by moral concern. The moral expansiveness framework also offers a less straightforward explanation for instrumental prosocial behavior, because instrumental helping explicitly involves self-oriented goals in addition to benefiting others. Although expanded moral concern may coexist with such behavior, it does not directly account for the instrumental motives underlying it. In this respect, the social-relational account may be more directly relevant to the positive direct association between nature connectedness and instrumental prosocial behavior.

### 4.2. The Associational Role of Authenticity in the Associations Between Nature Connectedness and General and Instrumental Prosocial Behavior

Beyond the associations discussed above, the present findings identify authenticity as a theoretically relevant associational variable in the differing relations of nature connectedness with general and instrumental prosocial behavior. Greater nature connectedness was associated with higher authenticity. After accounting for nature connectedness, authenticity was positively associated with general prosocial behavior but negatively associated with instrumental prosocial behavior. Correspondingly, the indirect association involving authenticity was positive for general prosocial behavior and negative for instrumental prosocial behavior. The positive association between nature connectedness and authenticity is consistent with the expanded-identity account, particularly its self-transcendent implications. Theoretically, a broadened sense of self that includes nature may correspond to closer alignment with personally meaningful values, consistent with evidence linking self-transcendence with authentic-self pursuit (Jiang & Sedikides, 2022). The present findings provide preliminary evidence that nature connectedness and authenticity are related during late childhood, although their temporal ordering cannot be established.

The positive association between authenticity and general prosocial behavior should not be interpreted as indicating that general prosocial behavior is necessarily altruistically motivated. As noted earlier, general prosocial measures capture helping, sharing, and caring without specifying the motives underlying these behaviors. This association is therefore consistent with previous evidence that general prosociality can broadly correspond with the authentic self and that authenticity is related to prosocial and constructive interpersonal tendencies (Jiang & Sedikides, 2022; Theran, 2021; Tou et al., 2015). Taken together, these associations suggest that greater authenticity may be related to a general tendency to engage positively with and respond to others without requiring any single underlying prosocial motive. A different pattern emerged for instrumental prosocial behavior. Its negative association with authenticity suggests that helping explicitly organized around self-oriented goals may be less closely aligned with authenticity. Such motives overlap conceptually with the self-presentational and externally oriented concerns associated with lower authenticity (Alchin et al., 2024; Jiang & Sedikides, 2022; Tou et al., 2015; Wood et al., 2008). Importantly, this pattern does not imply that instrumental prosocial behavior is inherently inauthentic; rather, its explicit self-oriented motivational component may make it less positively aligned with authenticity than general prosocial behavior.

This countervailing pattern also helps characterize the nonsignificant total association between nature connectedness and instrumental prosocial behavior. Although the total association was nonsignificant, a positive direct association emerged after authenticity was taken into account, whereas the indirect association involving authenticity was negative. A weak or nonsignificant total association does not necessarily imply the absence of meaningful component associations because direct and indirect associations with opposing signs may offset one another (Ledermann et al., 2025; MacKinnon et al., 2000; Rijnhart et al., 2021). Under the AVA framework, however, these components represent patterns of concurrent association rather than evidence of causal pathways. One hypothesis generated by this pattern is that the relation between nature connectedness and instrumental prosocial behavior may involve multiple, potentially countervailing processes: a social-relational tendency positively related to instrumental helping and an authenticity-related tendency negatively related to self-oriented helping. These possible processes were neither directly tested nor temporally ordered in the present study and therefore require confirmation through longitudinal or experimental research.

### 4.3. Theoretical and Practical Implications, Limitations, and Future Directions

The present study offers two theoretical implications. First, distinguishing the more specific construct of instrumental prosocial behavior from general prosocial behavior suggests that nature connectedness is not uniformly associated with prosociality; rather, its associations may vary according to the motivational characteristics of prosocial behavior. Second, authenticity emerged as a theoretically relevant associational variable, showing a positive association with general prosocial behavior but a negative association with instrumental prosocial behavior. This pattern raises the theoretical possibility that the association between nature connectedness and prosociality may involve multiple, potentially countervailing processes rather than a single uniform pathway, a proposition that requires longitudinal or experimental testing.

The findings also have practical relevance. Nature connectedness can be strengthened through meaningful engagement with natural environments, including direct experience, emotional involvement, and opportunities to develop a sense of meaning and identification with nature (Giusti et al., 2018; Lumber et al., 2017). Nature-based educational activities that encourage deeper connections with nature may therefore have potential benefits not only for environmental attitudes and psychological well-being but also for children’s social and self-development (Harris et al., 2025; Whitburn et al., 2023). In particular, as a possible application rather than a conclusion directly established by the present findings, activities that help children connect nature-related experiences with their sense of self and personally meaningful values might be explored as a means of supporting social engagement and authenticity. Given the cross-sectional design of the present study, this possibility remains preliminary and requires confirmation through longitudinal or experimental research.

Several limitations should be acknowledged. First, the cross-sectional design precludes causal or temporal conclusions, and unmeasured factors such as family socialization and personality may also contribute to the observed associations. Longitudinal and experimental studies are needed to clarify temporal relations and potential confounding influences. Second, all focal variables were assessed using self-report measures from a single informant, raising concerns about shared method variance and socially desirable responding. Moreover, the developmental appropriateness and cultural validity of some measures warrant further examination, particularly for Chinese children in late childhood. Future research should further validate these measures across age groups and cultural contexts while incorporating behavioral and multi-informant assessments. Third, the structural analyses treated the DIF-adjusted EAP factor scores as observed variables. Although this approach allowed the identified DIF to be incorporated into the factor scores, it did not explicitly account for factor-score uncertainty when estimating the structural associations and their standard errors. Future research could use joint measurement–structural models or other approaches that explicitly incorporate uncertainty in estimated factor scores. Fourth, the proposed social-relational process was not directly measured, and the present interpretation should therefore be regarded as tentative. Future studies should directly assess relevant interpersonal processes alongside authenticity. Fifth, the convenience sample was drawn from only two urban primary schools, and detailed socioeconomic information, environmental exposure, and broader contextual variation were not assessed. These features limit the generalizability of the findings and warrant replication in larger, more diverse samples and cultural settings, particularly given that the meaning of authenticity and responses to external influence may vary across cultural contexts. In addition, participants were nested within eight classrooms in two schools, but the small number of higher-level clusters limited our ability to account reliably for classroom- and school-level dependence. Finally, the general prosocial behavior measure did not specify the motives underlying helping, limiting conclusions about the motivational processes involved. The finding that the positive direct association between nature connectedness and instrumental prosocial behavior was evident among boys but not girls should also be interpreted cautiously and requires replication in larger and more diverse samples.

## 5. Conclusions

The present study extends research on nature connectedness and prosociality by distinguishing the more specific construct of instrumental prosocial behavior from general prosocial behavior. Nature connectedness was positively associated with general prosocial behavior and authenticity, whereas authenticity showed contrasting associations with general and instrumental prosocial behavior. For instrumental prosocial behavior, the nonsignificant overall association reflected opposing direct and indirect associations, with the positive direct association evident primarily among boys. Taken together, these findings are compatible with an expanded-identity account and highlight authenticity as a potentially important correlate in understanding how nature connectedness is differentially associated with general and instrumental prosocial behavior during late childhood.

## Figures and Tables

**Table 1:** Means, standard deviations, and correlations among study variables (n = 281).

Variable		*M*	*SD*	1	2	3	4	5
1.	Sex	0.47	0.50	—	0.16 **	−0.06	0.14 *	0.06
2.	Nature connectedness	4.18	0.82	0.17 **	—	0.27 ***	0.41 ***	0.07
3.	Authenticity	3.93	0.71	−0.06	0.28 ***	—	0.24 ***	−0.23 ***
4.	General prosocial behavior	2.58	0.38	0.12 *	0.40 ***	0.27 ***	—	−0.01
5.	Instrumental prosocial behavior	2.30	1.06	0.04	0.07	−0.21 ***	0.00	—

*Note*. Sex was dummy coded (0 = boy, 1 = girl). Correlations involving sex are point-biserial correlations. The lower triangle presents correlations based on raw mean scores, while the upper triangle presents correlations based on DIF-adjusted latent factor scores estimated using the EAP method. * *p* < 0.05, ** *p* < 0.01, *** *p* < 0.001.

**Table 2:** Path Coefficients for the Associational Variable Model.

Predictor	Authenticity				General Prosocial Behavior				Instrumental Prosocial Behavior			
	*B*	*SE*	β	*p*	*B*	*SE*	β	*p*	*B*	*SE*	β	*p*
Model 1: Total Association Analysis												
Nature connectedness	0.19	0.05	0.27	<0.001	0.41	0.06	0.41	<0.001	0.08	0.07	0.07	0.24
Model 2: Indirect Association Analysis												
Nature connectedness	0.19	0.05	0.27	<0.001	0.37	0.07	0.37	<0.001	0.16	0.07	0.15	0.02
Authenticity	—	—	—	—	0.19	0.09	0.14	0.03	−0.40	0.09	−0.27	<0.001
*R* ^2^	0.07				0.18				0.07			

*Note. B* = unstandardized regression coefficient; *SE* = standard error; β = standardized regression coefficient. Model 1 reports the path from nature connectedness to authenticity (a path) and the total associations of nature connectedness on general and instrumental prosocial behavior; Model 2 reports the indirect association path model based on DIF-adjusted EAP factor scores.

## Data Availability

The data are not publicly available due to privacy and ethical restrictions involving minors and children. De-identified data may be made available from the corresponding author upon reasonable request and subject to approval by the relevant ethics committee and administrative authorities.

## Abbreviations

The following abbreviations are used in this manuscript:

AVA	Associational Variable Analysis
CFA	Confirmatory Factor Analysis
CFI	Comparative Fit Index
CI	Confidence Interval
DIF	Differential Item Functioning
EAP	Expected A Posteriori
MNLFA	Moderated Nonlinear Factor Analysis
RMSEA	Root Mean Square Error of Approximation
SRMR	Standardized Root Mean Square Residual
TLI	Tucker–Lewis index

## Appendix A

**Table A1:** Results of the Regularized MNLFA Analyses.

**Panel A. *Model-Selection Summary***					
**Scale/Subscale**	**Optimal *τ***	**BIC**	**Uniform DIF, *n***	**Nonuniform DIF, *n***	**Total Nonzero DIF Parameters**
General prosocial behavior	3.925	5436.096	7	7	14
Instrumental prosocial behavior	29.842	9770.842	0	0	0
Nature connectedness	58.374	55,846.927	0	0	0
Self-alienation	56.706	30,885.010	0	0	0
Accepting external influence	28.183	18,871.507	0	0	0
Authentic living	19.296	12,166.042	1	0	1
**Panel B. *Nonzero DIF Parameters Retained by LASSO***					
**Scale/Subscale**	**Item**	**Parameter**	**Background** **Variable**	**Penalized** **Estimate**	**DIF Type**
General prosocial behavior	2	Intercept	Grade	0.040	Uniform
	3	Intercept	Grade	−0.017	Uniform
	3	Intercept	Sex	0.012	Uniform
	4	Intercept	Grade	0.036	Uniform
	4	Intercept	Sex	0.0147	Uniform
	5	Intercept	Grade	−0.003	Uniform
	5	Intercept	Sex	−0.010	Uniform
General prosocial behavior	1	Loading	Grade	−0.041	Nonuniform
	1	Loading	Sex	−0.031	Nonuniform
	2	Loading	Grade	0.016	Nonuniform
	3	Loading	Grade	−0.062	Nonuniform
	3	Loading	Sex	−0.040	Nonuniform
	4	Loading	Grade	−0.118	Nonuniform
	5	Loading	Sex	0.008	Nonuniform
Authentic living	8	Intercept	Sex	−0.097	Uniform

*Note.* Sex was effect-coded as boys = −1 and girls = 1; grade was effect-coded as Grade 4 = −1 and Grade 5 = 1. Predictors were internally standardized by regDIF before estimation. Counts in Panel A refer to nonzero parameters retained by LASSO. Intercept and loading effects represent uniform and nonuniform DIF, respectively. Estimates are penalized LASSO coefficients from the minimum-BIC solution and should not be interpreted as conventional unpenalized estimates or statistical significance tests. Values of τ and BIC are scale specific and should not be compared across scales. Blank entries in the Scale/Subscale column indicate the same scale as the preceding row. BIC = Bayesian information criterion; DIF = differential item functioning; LASSO = least absolute shrinkage and selection operator; MNLFA = moderated nonlinear factor analysis.

## References

[B1-behavsci-16-01785] Alchin C. E., Machin T. M., Martin N., Burton L. J. (2024). Authenticity and inauthenticity in adolescents: A scoping review. Adolescent Research Review.

[B2-behavsci-16-01785] Arbuthnott K. D. (2023). Nature exposure and social health: Prosocial behavior, social cohesion, and effect pathways. Journal of Environmental Psychology.

[B3-behavsci-16-01785] Barragan-Jason G., Loreau M., de Mazancourt C., Singer M. C., Parmesan C. (2023). Psychological and physical connections with nature improve both human well-being and nature conservation: A systematic review of meta-analyses. Biological Conservation.

[B4-behavsci-16-01785] Bauer D. J., Belzak W. C. M., Cole V. T. (2020). Simplifying the assessment of measurement invariance over multiple background variables: Using regularized moderated nonlinear factor analysis to detect differential item functioning. Structural Equation Modeling: A Multidisciplinary Journal.

[B5-behavsci-16-01785] Bayır-Toper A., Sellman E., Joseph S. (2022). Being yourself for the ‘greater good’: An empirical investigation of the moderation effect of authenticity between self-compassion and compassion for others. Current Psychology.

[B6-behavsci-16-01785] Behrens B., Edler K., Valentino K. (2025). Assessing whether negative parenting cognitions bias parent report of preschoolers’ externalizing symptoms: A regularized moderated non-linear factor analysis approach. Research on Child and Adolescent Psychopathology.

[B7-behavsci-16-01785] Belzak W. C. M. (2023). The regDIF R package: Evaluating complex sources of measurement bias using regularized differential item functioning. Structural Equation Modeling: A Multidisciplinary Journal.

[B8-behavsci-16-01785] Belzak W. C. M., Bauer D. J. (2020). Improving the assessment of measurement invariance: Using regularization to select anchor items and identify differential item functioning. Psychological Methods.

[B9-behavsci-16-01785] Boxer P., Tisak M. S., Goldstein S. E. (2004). Is it bad to be good? An exploration of aggressive and prosocial behavior subtypes in adolescence. Journal of Youth and Adolescence.

[B10-behavsci-16-01785] Böckler A., Tusche A., Singer T. (2016). The structure of human prosociality: Differentiating altruistically motivated, norm motivated, strategically motivated, and self-reported prosocial behavior. Social Psychological and Personality Science.

[B11-behavsci-16-01785] Brislin R. W. (1970). Back-translation for cross-cultural research. Journal of Cross-Cultural Psychology.

[B12-behavsci-16-01785] Carlo G., Padilla-Walker L. (2020). Adolescents’ prosocial behaviors through a multidimensional and multicultural lens. Child Development Perspectives.

[B13-behavsci-16-01785] Cheng J. C.-H., Monroe M. C. (2012). Connection to nature: Children’s affective attitude toward nature. Environment and Behavior.

[B14-behavsci-16-01785] Cirimele F., Pastorelli C., Bornstein M. H., Zuffianò A., Remondi C., Gerbino M., Bacchini D., Di Giunta L., Oburu P., Skinner A. T., Sorbring E., Steinberg L., Uribe Tirado L. M., Yotanyamaneewong S., Alampay L. P., Al-Hassan S. M., Chang L., Deater-Deckard K., Dodge K. A., Lansford J. E. (2025). Prosocial behavior and school performance in the transition to adolescence: A multicultural study. Applied Developmental Science.

[B15-behavsci-16-01785] Clayton S., Clayton S., Opotow S. (2003). Environmental identity: A conceptual and an operational definition. Identity and the natural environment: The psychological significance of nature.

[B16-behavsci-16-01785] Crimston C. R., Bain P. G., Hornsey M. J., Bastian B. (2016). Moral expansiveness: Examining variability in the extension of the moral world. Journal of Personality and Social Psychology.

[B17-behavsci-16-01785] Crone E. A., Achterberg M. (2022). Prosocial development in adolescence. Current Opinion in Psychology.

[B18-behavsci-16-01785] Davis N., Gatersleben B. (2013). Transcendent experiences in wild and manicured settings: The influence of the trait “connectedness to nature”. Ecopsychology.

[B19-behavsci-16-01785] Du Y., Kou J., Coghill D. (2008). The validity, reliability and normative scores of the parent, teacher and self report versions of the Strengths and Difficulties Questionnaire in China. Child and Adolescent Psychiatry and Mental Health.

[B20-behavsci-16-01785] Eagly A. H. (2009). The his and hers of prosocial behavior: An examination of the social psychology of gender. American Psychologist.

[B21-behavsci-16-01785] Eisenberg N., Spinrad T. L., Knafo-Noam A., Lamb M. E., Lerner R. M. (2015). Prosocial development. Handbook of child psychology and developmental science: Socioemotional processes.

[B22-behavsci-16-01785] Eisenberg N., VanSchyndel S. K., Spinrad T. L. (2016). Prosocial motivation: Inferences from an opaque body of work. Child Development.

[B23-behavsci-16-01785] Findley-Van Nostrand D., Ojanen T. (2018). Forms of prosocial behaviors are differentially linked to social goals and peer status in adolescents. The Journal of Genetic Psychology.

[B24-behavsci-16-01785] Giusti M., Svane U., Raymond C. M., Beery T. H. (2018). A framework to assess where and how children connect to nature. Frontiers in Psychology.

[B25-behavsci-16-01785] Goldy S. P., Piff P. K. (2020). Toward a social ecology of prosociality: Why, when, and where nature enhances social connection. Current Opinion in Psychology.

[B26-behavsci-16-01785] Goodman R., Meltzer H., Bailey V. (1998). The strengths and difficulties questionnaire: A pilot study on the validity of the self-report version. European Child & Adolescent Psychiatry.

[B27-behavsci-16-01785] Grueneisen S., Warneken F. (2022). The development of prosocial behavior—From sympathy to strategy. Current Opinion in Psychology.

[B28-behavsci-16-01785] Hamburg D. A., Hamburg B. A. (2004). Learning to live together: Preventing hatred and violence in child and adolescent development.

[B29-behavsci-16-01785] Harris N. M., Hartwell B., Thomas L., Grace M. (2025). How can nature connectedness and behaviours for learning be deliberately developed in children, adolescents and young adults? A systematic literature review. Child and Adolescent Mental Health.

[B30-behavsci-16-01785] Hepach R., Daniel E. (2026). How developing prosocial motivations shape children’s altruism. Trends in Cognitive Sciences.

[B31-behavsci-16-01785] Hirani S., Ojukwu E., Bandara N. A. (2022). Understanding the role of prosocial behavior in youth mental health: Findings from a scoping review. Adolescents.

[B32-behavsci-16-01785] Hughes J., Richardson M., Lumber R. (2018). Evaluating connection to nature and the relationship with conservation behaviour in children. Journal for Nature Conservation.

[B33-behavsci-16-01785] Ives C. D., Giusti M., Fischer J., Abson D. J., Klaniecki K., Dorninger C., Laudan J., Barthel S., Abernethy P., Martín-López B., Raymond C. M., Kendal D., von Wehrden H. (2017). Human–nature connection: A multidisciplinary review. Current Opinion in Environmental Sustainability.

[B34-behavsci-16-01785] Jiang T., Sedikides C. (2022). Awe motivates authentic-self pursuit via self-transcendence: Implications for prosociality. Journal of Personality and Social Psychology.

[B35-behavsci-16-01785] Kernis M. H., Goldman B. M., Zanna M. P. (2006). A multicomponent conceptualization of authenticity: Theory and Research. Advances in experimental social psychology.

[B36-behavsci-16-01785] Kline R. B. (2016). Principles and practice of structural equation modeling.

[B37-behavsci-16-01785] LaFontana K. M., Cillessen A. H. N. (2010). Developmental changes in the priority of perceived status in childhood and adolescence. Social Development.

[B38-behavsci-16-01785] Ledermann T., Rudaz M., Fritz M. S. (2025). On partial versus full mediation and the importance of effect sizes. Advances in Methods and Practices in Psychological Science.

[B39-behavsci-16-01785] Lumber R., Richardson M., Sheffield D. (2017). Beyond knowing nature: Contact, emotion, compassion, meaning, and beauty are pathways to nature connection. PLoS ONE.

[B40-behavsci-16-01785] MacKinnon D. P., Krull J. L., Lockwood C. M. (2000). Equivalence of the mediation, confounding and suppression effect. Prevention Science.

[B41-behavsci-16-01785] Magro S. W., Berry D., Palmer A. R., Roisman G. I. (2026). Teacher–student relationship quality and social, academic, and behavioral adjustment are associated within and between persons from kindergarten to grade 6. Developmental Psychology.

[B42-behavsci-16-01785] Magro S. W., DeJoseph M. L., Pianta R. C., Roisman G. I. (2024). Using moderated nonlinear factor models to adjust for differential item functioning in the Student-Teacher Relationship Scale from kindergarten to Grade 6. Journal of School Psychology.

[B43-behavsci-16-01785] Malti T., Dys S. P. (2018). From being nice to being kind: Development of prosocial behaviors. Current Opinion in Psychology.

[B44-behavsci-16-01785] Maniaci M. R., Rogge R. D. (2014). Caring about carelessness: Participant inattention and its effects on research. Journal of Research in Personality.

[B45-behavsci-16-01785] Martin A., Olson K. R. (2015). Beyond good and evil: What motivations underlie children’s prosocial behavior?. Perspectives on Psychological Science.

[B46-behavsci-16-01785] Martin C., Czellar S. (2016). The extended inclusion of nature in self scale. Journal of Environmental Psychology.

[B47-behavsci-16-01785] Mayer F. S., Frantz C. M. (2004). The connectedness to nature scale: A measure of individuals’ feeling in community with nature. Journal of Environmental Psychology.

[B48-behavsci-16-01785] Mei D., Yang D., Li T., Zhang X., Rao K., Li L. M. W. (2024). Nature contact promotes prosociality: The mediating roles of self-transcendence, nature connectedness, and materialism. Journal of Environmental Psychology.

[B49-behavsci-16-01785] Memmott-Elison M. K., Holmgren H. G., Padilla-Walker L. M., Hawkins A. J. (2020). Associations between prosocial behavior, externalizing behaviors, and internalizing symptoms during adolescence: A meta-analysis. Journal of Adolescence.

[B50-behavsci-16-01785] Mouratidis A., Sayil M., Kumru A., Selcuk B., Soenens B. (2019). Maternal knowledge as a mediator of the relation between maternal psychological control and altruistic prosocial, instrumental prosocial, and antisocial behavior. Merrill-Palmer Quarterly.

[B51-behavsci-16-01785] Nisbet E. K., Zelenski J. M., Murphy S. A. (2009). The nature relatedness scale: Linking individuals’ connection with nature to environmental concern and behavior. Environment and Behavior.

[B52-behavsci-16-01785] Oberle E., Ji X. R., Molyneux T. M. (2023). Pathways from prosocial behaviour to emotional health and academic achievement in early adolescence. The Journal of Early Adolescence.

[B53-behavsci-16-01785] Olivos P., Clayton S. (2017). Self, nature and well-being: Sense of connectedness and environmental identity for quality of life. Handbook of environmental psychology and quality of life research.

[B54-behavsci-16-01785] Penner L. A., Dovidio J. F., Piliavin J. A., Schroeder D. A. (2005). Prosocial behavior: Multilevel perspectives. Annual Review of Psychology.

[B55-behavsci-16-01785] Pritchard A., Richardson M., Sheffield D., McEwan K. (2020). The relationship between nature connectedness and eudaimonic well-being: A meta-analysis. Journal of Happiness Studies.

[B56-behavsci-16-01785] R Core Team (2026). R: A language and environment for statistical computing.

[B57-behavsci-16-01785] Revelle W. (2026). psych: Procedures for psychological, psychometric, and personality research *(R package version 2.6.4)*.

[B58-behavsci-16-01785] Rijnhart J. J. M., Lamp S. J., Valente M. J., MacKinnon D. P., Twisk J. W. R., Heymans M. W. (2021). Mediation analysis methods used in observational research: A scoping review and recommendations. BMC Medical Research Methodology.

[B59-behavsci-16-01785] Rosseel Y. (2012). lavaan: An R package for structural equation modeling. Journal of Statistical Software.

[B60-behavsci-16-01785] Ruckert J. H., Granato H. F., Barnes A., Martin E. G. (2025). Nature relatedness and self-transcendent emotions: A mediational exploration of the role of mindfulness. The Humanistic Psychologist.

[B61-behavsci-16-01785] Salazar G., Kunkle K., Monroe M. C. (2020). Practitioner guide to assessing connection to nature.

[B62-behavsci-16-01785] Salazar G., Monroe M. C., Jordan C., Ardoin N. M., Beery T. H. (2021). Improving assessments of connection to nature: A participatory approach. Frontiers in Ecology and Evolution.

[B63-behavsci-16-01785] Schultz P. W., Schmuck P., Schultz W. P. (2002). Inclusion with nature: The psychology of human-nature relations. Psychology of sustainable development.

[B64-behavsci-16-01785] Sedikides C., Schlegel R. J. (2024). Distilling the concept of authenticity. Nature Reviews Psychology.

[B65-behavsci-16-01785] Singer P. (2011). The expanding circle: Ethics, evolution, and moral progress.

[B66-behavsci-16-01785] Song L., Wang Y., Zhao Y. (2020). Reliability and validity of the authenticity scale for Chinese adolescents. Chinese Journal of Clinical Psychology.

[B67-behavsci-16-01785] Stevens A. K., Janssen T., Belzak W. C. M., Treloar Padovano H., Jackson K. M. (2022). Comprehensive measurement invariance of alcohol outcome expectancies among adolescents using regularized moderated nonlinear factor analysis. Addictive Behaviors.

[B68-behavsci-16-01785] Tam K.-P. (2013). Concepts and measures related to connection to nature: Similarities and differences. Journal of Environmental Psychology.

[B69-behavsci-16-01785] Taylor L. K. (2020). The developmental peacebuilding model (DPM) of children’s prosocial behaviors in settings of intergroup conflict. Child Development Perspectives.

[B70-behavsci-16-01785] Theran S. A. (2021). The roles of authenticity in relationships and attachment in adolescents’ prosocial experiences. The Journal of Genetic Psychology.

[B71-behavsci-16-01785] Thomaes S., Sedikides C., van den Bos N., Hutteman R., Reijntjes A. (2017). Happy to be “me?” Authenticity, psychological need satisfaction, and subjective well-being in adolescence. Child Development.

[B72-behavsci-16-01785] Tou R. Y. W., Baker Z. G., Hadden B. W., Lin Y.-C. (2015). The real me: Authenticity, interpersonal goals, and conflict tactics. Personality and Individual Differences.

[B73-behavsci-16-01785] Weems C. F. (2025). An alternative framework for nonexperimental cross-sectional mediation studies: Associational variable analysis. Psychological Methods.

[B74-behavsci-16-01785] Whitburn J., Abrahamse W., Linklater W. (2023). Do environmental education fieldtrips strengthen children’s connection to nature and promote environmental behaviour or wellbeing?. Current Research in Ecological and Social Psychology.

[B75-behavsci-16-01785] Wickham H., Averick M., Bryan J., Chang W., McGowan L. D. A., François R., Grolemund G., Hayes A., Henry L., Hester J., Kuhn M., Pedersen T. L., Miller E., Bache S. M., Müller K., Ooms J., Robinson D., Seidel D. P., Spinu V., Yutani H. (2019). Welcome to the tidyverse. Journal of Open Source Software.

[B76-behavsci-16-01785] Wood A. M., Linley P. A., Maltby J., Baliousis M., Joseph S. (2008). The authentic personality: A theoretical and empirical conceptualization and the development of the authenticity scale. Journal of Counseling Psychology.

[B77-behavsci-16-01785] Yao Z., Li M., Liu H. (2025). Cultivating care through nature: A meta-analysis of nature connectedness and prosociality. Sustainability.

[B78-behavsci-16-01785] Yentes R., Wilhelm F. (2023). careless: Procedures for computing indices of careless responding *(R package version 1.2.2)*.

[B79-behavsci-16-01785] Yildirim G., Willison J., Bentley B. (2026). Nature connectedness framework for adolescents: An integrative review. Frontiers in Psychology.

[B80-behavsci-16-01785] Zhao X., Lynch J. G., Chen Q. (2010). Reconsidering Baron and Kenny: Myths and truths about mediation analysis. Journal of Consumer Research.

[B81-behavsci-16-01785] Zhou J., Hu Y., Liu Y., Song J., Tang J. (2026). Developmental cascades linking academic achievement, prosocial behavior, peer relationships and teacher–student relationships in Chinese early adolescents. Journal of Youth and Adolescence.

